# Severe Enoral Bleeding with a Direct Oral Anticoagulant after Tooth Extraction and Heparin Bridging Treatment

**DOI:** 10.1155/2019/6208604

**Published:** 2019-10-29

**Authors:** Simone Ehrhard, John Patrik Burkhard, Aristomenis K. Exadaktylos, Thomas C. Sauter

**Affiliations:** ^1^Department of Emergency Medicine, Inselspital University Hospital, Bern, Switzerland; ^2^Department of Cranio-Maxillofacial Surgery, Inselspital University Hospital, Bern, Switzerland; ^3^Skills Lab Lernzentrum, Charité Universitätsmedizin Berlin, Chariteplatz 1, Berlin 10117, Germany

## Abstract

**Background:**

The number of patients receiving direct oral anticoagulants (DOACs) is increasing, however, this treatment is associated with the risk of bleeding. More than 10 percent of patients on DOACs have to interrupt their anticoagulation for an invasive procedure every year. For this reason, the correct management of DOACs in the perioperative setting is mandatory.

**Case Presentation:**

An 81-year-old male patient, with known impaired renal function, presented to our emergency department with a severe enoral bleeding after tooth extraction. The DOAC therapy—indicated by known atrial fibrillation—was interrupted perioperatively and bridged with Low Molecular Weight Heparin (LMWH). The acute bleeding was stopped by local surgery. The factors contributing to the bleeding complication were bridging of DOAC treatment, together with prolonged drug action in chronic kidney disease.

**Conclusion:**

In order to decide whether it is necessary to stop DOAC medication for tooth extraction, it is important to carefully weigh up the individual risks of bleeding and thrombosis. If DOAC therapy is interrupted, bridging should be reserved for thromboembolic high-risk situations. Particular caution is required in patients with impaired kidney function, due to the risk of accumulation and prolonged anticoagulant effect of both DOACs and LMWH.

## 1. Introduction

Direct oral anticoagulants (DOACs) were introduced in recent years and are of increasing importance in everyday clinical work, in both emergency departments (EDs) and general practice [1, 2]. Excessive bleeding is a risk for patients on anticoagulant therapy. Hemorrhage after tooth extraction is frequent with an incidence up to 26% and may range from minor to life threatening [3]. Management of DOACs in the perioperative setting required the responsible physician to have an understanding of DOAC indications, pharmacokinetics, drug-drug interactions, and their effects on laboratory assays [4, 5]. More than 10% of all patients on DOACs have to interrupt their anticoagulant medication before an invasive procedure every year [4, 6, 7]. Decisions about the time to stop DOACs preoperatively must be based on the half-life of the prescribed anticoagulant therapy, the bleeding risk of the invasive procedure, and the bleeding (HAS-BLED score) respectively thrombo-embolic (CHADS2 score) risk to the patient [4, 8]. To guide the perioperative use of DOACs, a checklist should be available which covers all aspects of the planned surgical procedure as well as lists the patient-specific characteristics that may increase the risks of bleeding or thrombosis [7]. For this purpose, specific guidance was developed to manage dental patients taking anticoagulants or antiplatelet drugs [9]. Historically the application of LMWH after stopping oral anticoagulants was recommended to avoid a perioperative gap with insufficient anticoagulation [8]. There is no current literature to support the practice of heparin bridging to reduce the perioperative risk of thromboembolism at DOAC arrest [7]. On the contrary, some studies demonstrated even a higher bleeding risk without any reduction in thromboembolic complications in patients taking DOACs or vitamin K antagonists when receiving bridging treatment [10]. DOACs have a shorter elimination half-life than most vitamin K antagonists and therefore heparin bridging has no clinical benefit in patients with a short period of perioperative DOAC interruption [7]. In summary, this suggests that peri-operative bridging treatment should be restricted to patients with high thromboembolic risk and prolonged DOAC arrest (>72–96 hours) [10]. Whereas the cessation of a vitamin K antagonist induces an imbalance between pro- and anticoagulatory factors, making bridging therapy more important, this phenomenon is not present with DOACs. Nevertheless, many physicians still continue to bridge patients during interruption of DOAC therapy [11].

## 2. Case Presentation

An 81-year-old male patient was admitted from a nursing home to our ED because of severe enoral bleeding after the extraction of five teeth from the right lower and upper jaw, performed on the previous day in an outpatient clinic. Due to atrial fibrillation (AF), the patient was under oral anticoagulation with apixaban, 2.5 mg twice daily. His CHADS_2_ score showed two points (age and hypertension), leading to an intermediate stroke risk of 4.0% per year [12]. DOAC medication was interrupted five days before the planned dental surgery. Bridging therapy with the subcutaneous injection of 0.8 ml nadroparin per day was established two days after oral anticoagulation was interrupted. The last dose of nadroparin was administered more than 24 hours before surgery.

The patient had a history of lower gastrointestinal bleeding and no co-medication with nonsteroidal or corticosteroidal medication. Renal function was moderately impaired (KDIGO G2), but no creatinine >200 *µ*mol/l and no history or acute evidence of uncontrolled hypertension >160 mmHg were present, as requested in the HAS-BLED score. This led to a HAS-BLED score of 2 (1 point for age >65 years, 1 point for previous bleeding), corresponding to a bleeding risk of 4.1% per year in a validation study of the HAS-BLED score [13].

In addition to atrial fibrillation and chronic renal insufficiency, the patient was also known to have severe aortic stenosis, coronary and hypertensive cardiopathy, and Parkinson's syndrome. His daily medications were metoprolol, torasemide, aldactone, atorvastatin, citalopram, levodopamine, finasteride, pantoprazole, mesalazine, and apixaban as mentioned above.

**Figure 1:**
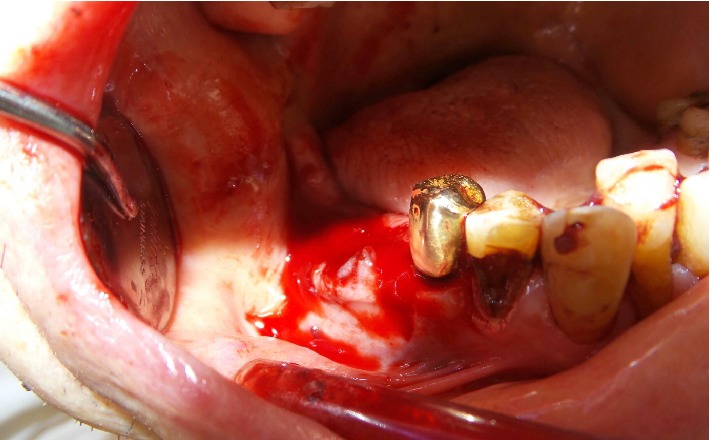
Oral bleeding after removal of the first and second molars.

On admission the patient was hypotonic (initial vital signs: blood pressure: 102/67 mmHg, pulse: 60 bpm on beta-blockers) and afebrile (36.6°C) with a documented body weight of 74.8 kg. The clinical examination of heart, lungs, and abdomen was normal. Further examination revealed enoral ecchymosis of the right cheek and a total of five extraction sockets in the upper and lower jaw. In the mandible, the first and second molars (46, 47) were removed. The oral mucosa in this area was swollen and partially adapted to the alveolar bone with sutures. After initial removal of large blood clots, active bleeding was observed in the region of the extraction sockets 46 and 47 (Figure 1). Laboratory values on admission revealed a haemoglobin of 122 g/L, a platelet count of 249 G/L, and a creatinine of 142 *µ*mol/l (eGFR 41 ml/min). Coagulation tests and drug levels are displayed in Table 1.

**Figure 2:**
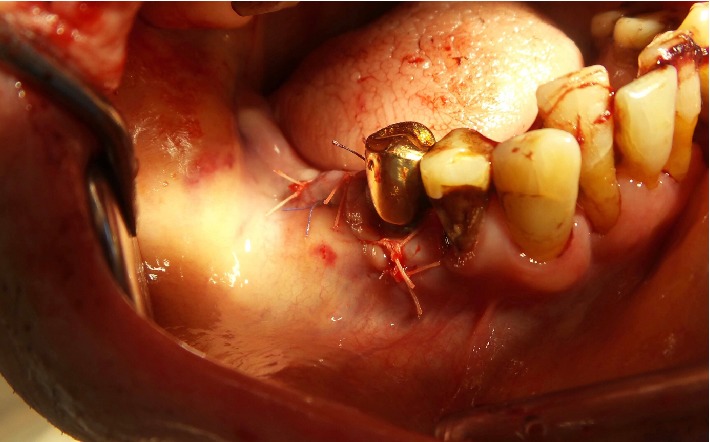
Wound condition after haemostasis and closure with a vestibular mucoperiosteal flap.

Initial treatment consisted of a fluid bolus and a gauze pack soaked with tranexamic acid, with pressure directly over the enoral wounds assisted by the patient biting down on the gauze. Despite these local measures, bleeding from the extraction site continued. To ensure sustained haemostasis, local flap surgery was performed by the craniomaxillofacial surgeons. Local anesthesia (Articaine 4% with 1 : 100,000 epinephrine) was performed vestibular to the alveolar ridge. The tooth extraction socket was curetted, all blood clots and debris removed and rinsed with saline solution. To support local haemostasis, several absorbable gelatin sponges with topical thrombin (Spongostan®, Ethicon, Germany) were inserted into the bony socket. A vestibular mucoperiosteal flap was created to cover the extraction wound. A long-lasting absorbable suture (Vicryl 4-0, Ethicon Inc.) was used to achieve primary wound closure (Figure 2). According to our local guidelines, no systemic reversal of anticoagulant therapy was performed, as the bleeding was controlled by surgical intervention [7].

During surveillance in the ED and the further hospitalisation, the patient remained haemodynamically stable. Haemoglobin dropped to 84 g/l at 24 hours after presentation and remained stable until discharge to the nursing home 3 days after admission. Oral anticoagulation with apixaban was restarted 4 days after the bleeding event with the same dose.

## 3. Discussion

The management of spontaneous bleedings under oral anticoagulation and the perioperative management of patients under DOAC therapy are essential. Specific scores exist to assist with assessing the AF-related thromboembolic risk (CHADS_2_ score) in the absence of anticoagulation, and the risk of major bleeding (HAS-BLED score) in patients with oral anticoagulant treatment [7]. However, the utility of these scores has not been prospectively validated in the perioperative setting [7, 14]. Moreover, each operation should be classified according to the bleeding risk [6]. The bleeding risk of tooth extraction is minimal when limited up to three teeth [6]. There is evidence that continuation of DOAC therapy is safe for minor dental procedures [15]. When DOAC therapy is stopped for surgery, as in the case we present, bridging with LMWH is only recommended for patients with a high risk of thromboembolism [8].

In each situation, the risk of bleeding must be weighed against the risk of a thromboembolic event. Assessing the risk for bleeding under anticoagulation, with a HES-BLED score of 2 and a low to moderate procedural bleeding risk, the cumulative bleeding risk for this patient was only moderately elevated. The risk for a thromboembolic event was elevated with a CHADS_2_ score of 2, but with no recent thromboembolism in the past 12 months and impaired renal function, stopping apixaban prior to the tooth extraction without bridging would have been appropriate according to international guidelines [16].

When the thromboembolic risk outweighs the risk of bleeding, heparin bridging should be discussed [7]. For LMWH, the dose and regimen must be adapted according to the patient's clinical characteristics (e.g., weight and renal function) taking into account the risk of bleeding [17]. In contrast to subcutaneously administered LMWH, the use of intravenous unfractionated heparin (UFH) requires hospitalization in order to monitor anticoagulant level, but has the advantage of being eliminated independently of the patient's renal function [11]. In addition, UFH can be completely antagonized in contrast to LMWH [11]. In any case, the decision of perioperative management of anticoagulant therapy should be made in consultation of the individual patient taking into account of all associated circumstances.

The renal function in this patient was impaired and led to a persistently elevated anti-Xa level at hospital admission, even though the last dose of apixaban was administered >5 days previously and the last injection of LMWH >24 hours prior to admission. Apixaban is mainly metabolized via CYP3A4 in the liver and only partially excreted by the kidneys, making apixaban a reasonable choice in patients with limited renal function. Nevertheless, the anti-Xa level of this patient was elevated, which emphasizes the need for special caution in patients with impaired kidney function even for apixaban. Two multicenter studies concluded that 48 hours without DOAC treatment might not guarantee the absence of residual anticoagulant effect at the time of intervention in up to 15% of all patients [18, 19]. Together with the high inter-individual variability of plasma concentrations, it is now suggested that the ideal timing of stopping DOAC treatment is based on plasma concentration measurements of DOACs in the periprocedural setting, especially for procedures with a high risk of bleeding [19, 20].

When acute bleeding occurs, rapid assessment of a patient's anticoagulation level helps to determine the anticoagulant's contribution to the bleeding, the need for a reversal strategy, and to assist in planning the time of invasive surgery, if required [5]. As the anticoagulant activity of DOACs is directly proportional to the plasma concentration, a direct measurement by liquid chromatography/tandem mass spectrometry would be the most accurate way to measure drug concentrations [21]. Unfortunately, this technology is not available in most acute care settings. The use of prothrombin time (PT) and activated partial thromboplastin time (aPTT) assays only poorly reflect the anticoagulant effect of apixaban [21]. For this reason, the best measurement for assessing the effect of apixaban is a calibrated anti-Xa-activity assay. LMWH is also monitored with anti-Xa activity measurements [5]. In our patient, post-extraction measurements of drug activity did not help to guide therapy, as the anti-Xa-activity assay available in our hospital used for the apixaban level is influenced by the given LMWH and vice versa. These results of the anti-Xa assays could only indicate that anticoagulant activity caused by LMWH and/or apixaban was present on admission. Therefore, especially in emergency medicine with urgent treatment indications, it is important to be aware that interferences between LMWH and DOAC activity measurement may generally lead to elevated drug levels with limited validity for a particular medication. Elevated drug levels indicate a haemostatic derangement in general and incorrect interpretation of these results may lead to dangerous patient management. Although apixaban was restarted in an unaltered dose at discharge from hospital, close monitoring in this patient was recommended to avoid accumulation.

Guidelines for bleeding under anticoagulation are important for ED clinicians. A previous study concluded that patients under DOACs and phenprocoumon with bleeding events after tooth extraction have a longer length of stay in the ED and more frequent surgical intervention than patients without anticoagulant therapy [22]. With the increasing number of different anticoagulation strategies in recent years, the training of emergency physicians as well as family doctors who prescribe and stop DOAC therapy perioperatively and the implementation of treatment algorithms have become increasingly important.

Therefore, in our institution (ED, Inselspital, University Hospital, Bern, Switzerland), a pragmatic treatment algorithm for bleeding in combination with anticoagulant treatment has been implemented [5].

## 4. Conclusion

In order to decide whether it is necessary to stop DOAC medication for tooth extraction, it is important to carefully weigh up the individual risk of bleeding and of thrombosis. If three teeth or fewer are extracted, the continuation of DOAC therapy can be safe. If DOAC therapy is interrupted, bridging should be reserved for thromboembolic high-risk situations. Particular caution is required in patients with renal insufficiency as it may lead to prolonged anticoagulant effect.

Since bleeding after tooth extraction is difficult to treat in anticoagulated patients, local treatment algorithms are essential for anticoagulation related bleeding.

## Figures and Tables

**Table 1:** Coagulation tests on emergency department admission.

Parameter	Norm values	On admission
INR	0.7–1.2	1.13
PT (s)	10–12	11.7
aPTT (s)	25–36	37.9
Thrombin time (s)	15.5–19.4	17.6
Fibrinogen (g/L)	1.75–3.75	3.19
Low molecular weight heparin (LMWH) anti-Xa (AntiXa/mL)	0.6–1.0 (at 4 hours after injection)	1.02 (at >24 hours after injection)
Apixaban anti-Xa (ng/mL)	No anticoagulant effect <30	64.89

Activated partial thromboplastin time (aPTT); International normalised ratio (INR); Prothrombin time (PT).
